# Supplementary material for: Lagrangian betweenness as a measure of bottlenecks in dynamical systems with oceanographic examples
Source: Nat Commun. 2021 Aug 16;12:4935. doi: 10.1038/s41467-021-25155-9 (PMC8368092; doi:10.1038/s41467-021-25155-9)
Supplement: Supplementary file 1 — Supplementary Information [file 41467_2021_25155_MOESM1_ESM.pdf]

Supplementary Figures for:
"Lagrangian betweenness as a measure of bottlenecks in dynamical systems with oceanographic examples"

Enrico Ser-Giacomi*
Department of Earth, Atmospheric and Planetary Sciences,
Massachusetts Institute of Technology, 54-1514 MIT, Cambridge, MA 02139, USA.

Alberto Baudena
Sorbonne Université, Institut de la Mer de Villefranche sur mer,
Laboratoire d'Océanographie de Villefranche, F-06230 Villefranche-sur-Mer, France

Vincent Rossi
Mediterranean Institute of Oceanography (UM110, UMR 7294) ;
CNRS, Aix Marseille Univ., Univ. Toulon, IRD; Marseille 13288, France

Mick Follows
Department of Earth, Atmospheric and Planetary Sciences,
Massachusetts Institute of Technology, 54-1514 MIT, Cambridge, MA 02139, USA.

Sophie Clayton
Old Dominion University, 4600 Elkhorn Ave, Norfolk, VA 23529, USA.

Ruggero Vasile
UP Transfer GmbH, Am Neuen Palais 10, 14469 Potsdam, Germany and
GFZ German Research Centre for Geosciences, Telegrafenberg, 14473 Potsdam, Germany

Cristóbal López and Emilio Hernández-García
IFISC (CSIC-UIB), Instituto de Física Interdisciplinar y Sistemas Complejos, E-07122 Palma de Mallorca, Spain

SUPPLEMENTARY INFORMATION FIGURES

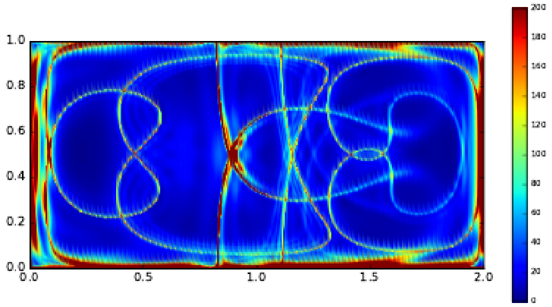

(a) B^L with $N = 2$

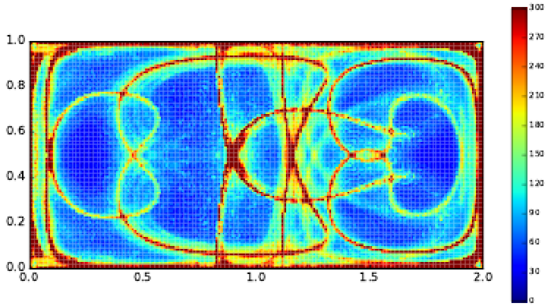

(b) $\bar{B}^{MPP}$ with $M = 2$

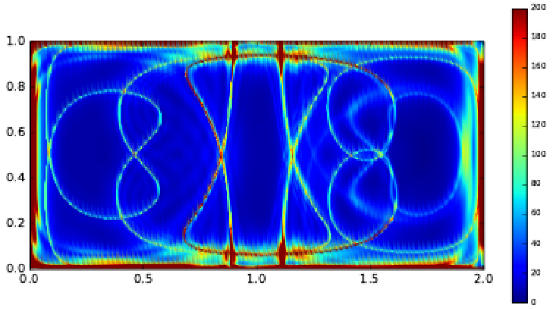

(c) B^L with $N = 3$

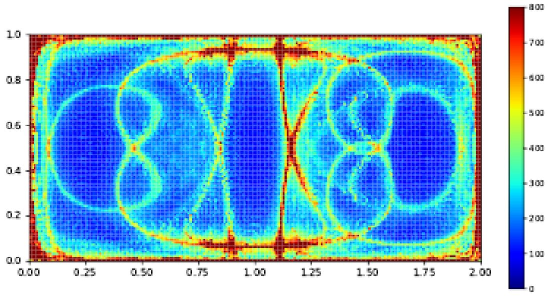

(d) $\bar{B}^{MPP}$ with $M = 3$

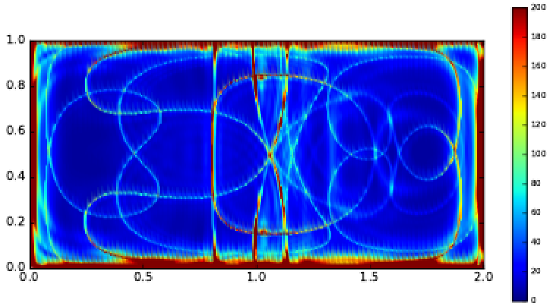

(e) B^L with $N = 5$

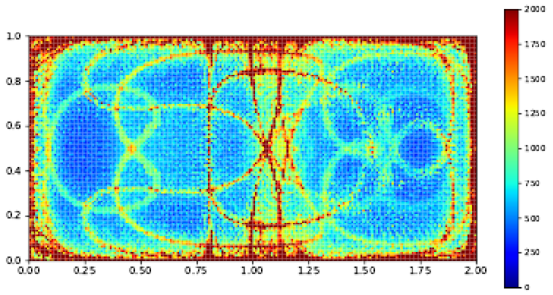

(f) $\bar{B}^{MPP}$ with $M = 5$

SUPPLEMENTARY FIG. 1: Betweenness for the double gyre flow. Panels (a), (c) and (e) show B^L calculated from Eq. (**??**) with $N = 2, 3, 5$ while panels (b), (d) and (f) show the symmetrized-in-time MPP-betweenness $\bar{B}^{MPP}$ of 2, 3 and 5 steps. The time interval is always $[0; 15]$, so that depending on the number of steps the duration of single steps is properly fixed to always match such time interval: therefore, for 2, 3 and 5 steps the step duration is Δt =7.5, 5 and 3 respectively.

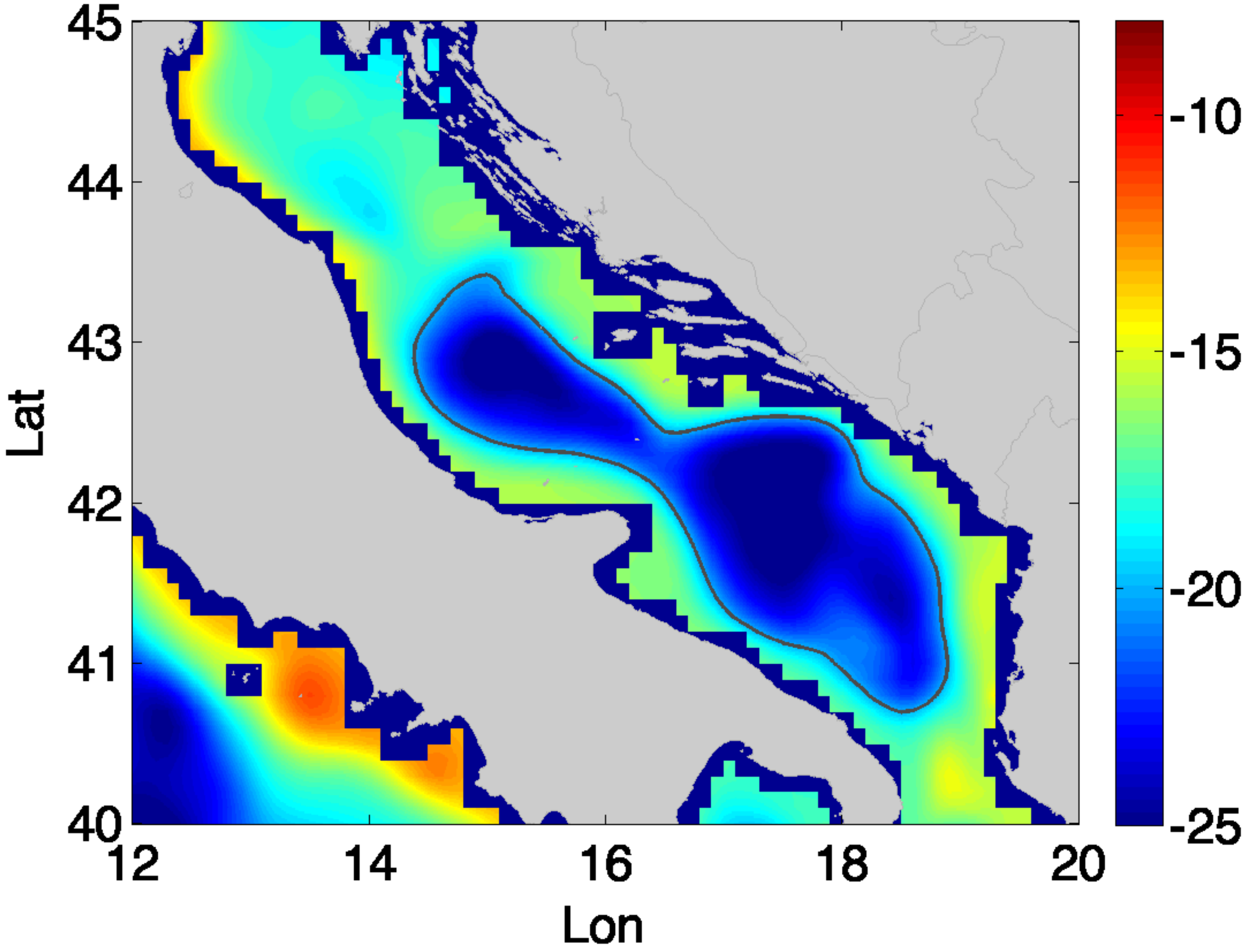

SUPPLEMENTARY FIG. 2: Sea surface height (SSH) from model data of the Adriatic Sea averaged from the 1st to the 15th of December 2013, expressed in *cm*. Under the geostrophic approximation, isolines of SSH are tangent to the direction of the currents. We clearly identify the cyclonic signature of the Middle and South Adriatic Gyres. The grey solid line is the -20 *cm* SSH isoline used as threshold for defining the interior of the two gyres.

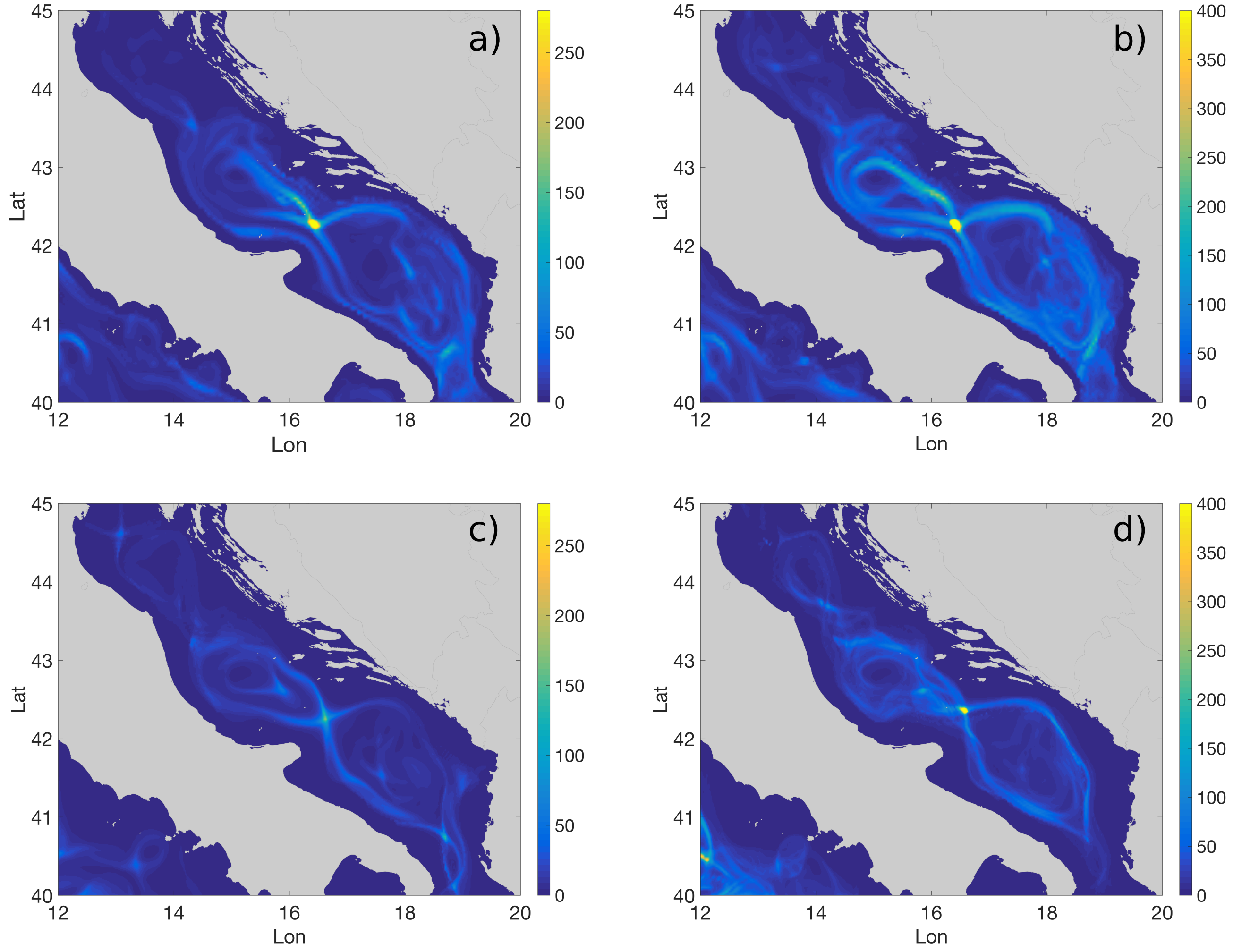

SUPPLEMENTARY FIG. 3: 'Betweenness in the Adriatic Sea. B^L fields calculated at the 1st of December 2013, for $\tau = 15$ and 30 days computed using the high resolution model velocity field (panel (a) and (b)) and the regional altimetry-derived velocity field (panel (c) and (d)). See Section Methods for details on the products used.

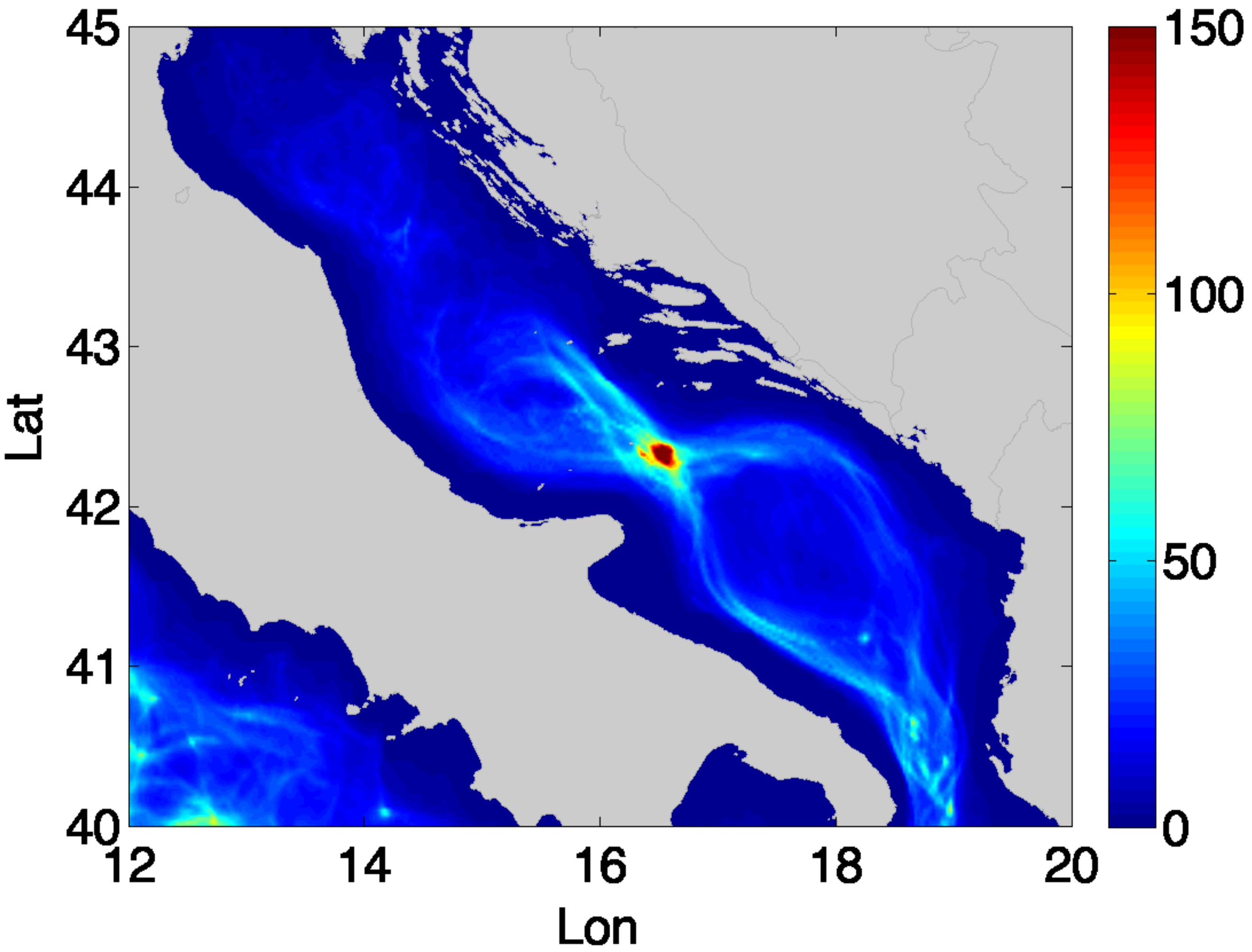

SUPPLEMENTARY FIG. 4: Time-average of the B^L field from regional altimetry-derived velocity field of the Adriatic Sea across the years 2002-2013, starting each calculation the 1st of December and with and integration time τ of 30 days. Strikingly, the position of the peak remained almost fixed across 12 years of observations.

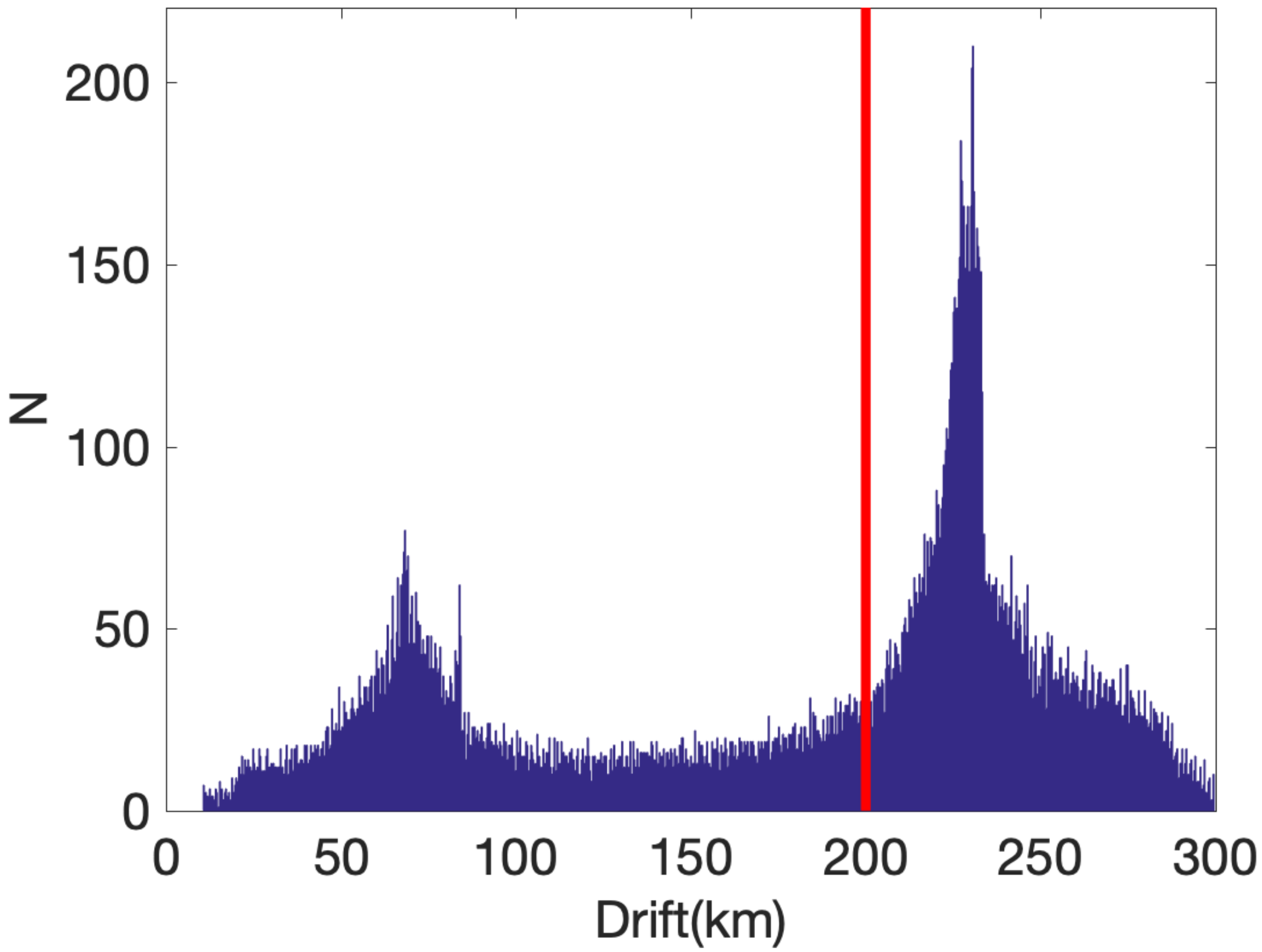

SUPPLEMENTARY FIG. 5: Histogram of 7 days backward-in-time Lagrangian drift distances of the ensemble of particles used for composing the patches of Fig. 7. The chosen threshold is equal to 200 km and is represented as a vertical red line. The strong bimodality of the drift distribution permits to clearly identify and separate the two water patches. Specifically, the patch presenting a drift larger than the threshold is associated directly to the Circumpolar Current (red color). Conversely, the patch of particles characterized by drifts smaller than than the threshold is associated to water coming from south-east of the Polar Front (white color).

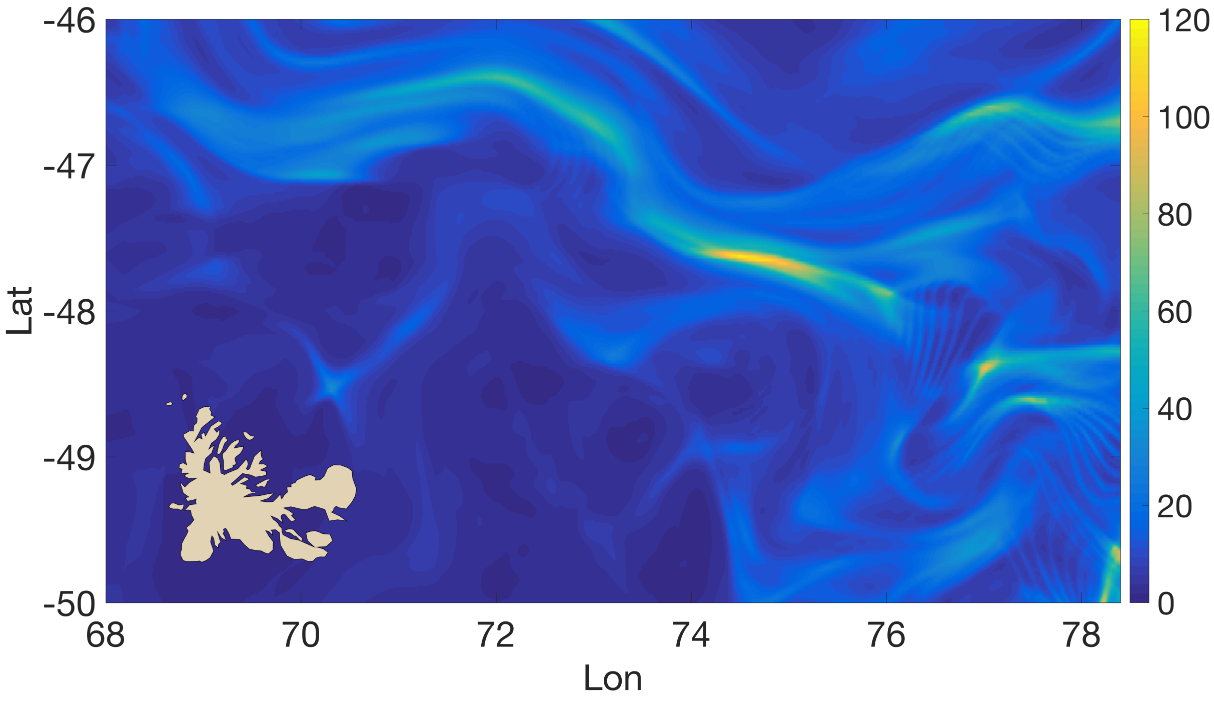

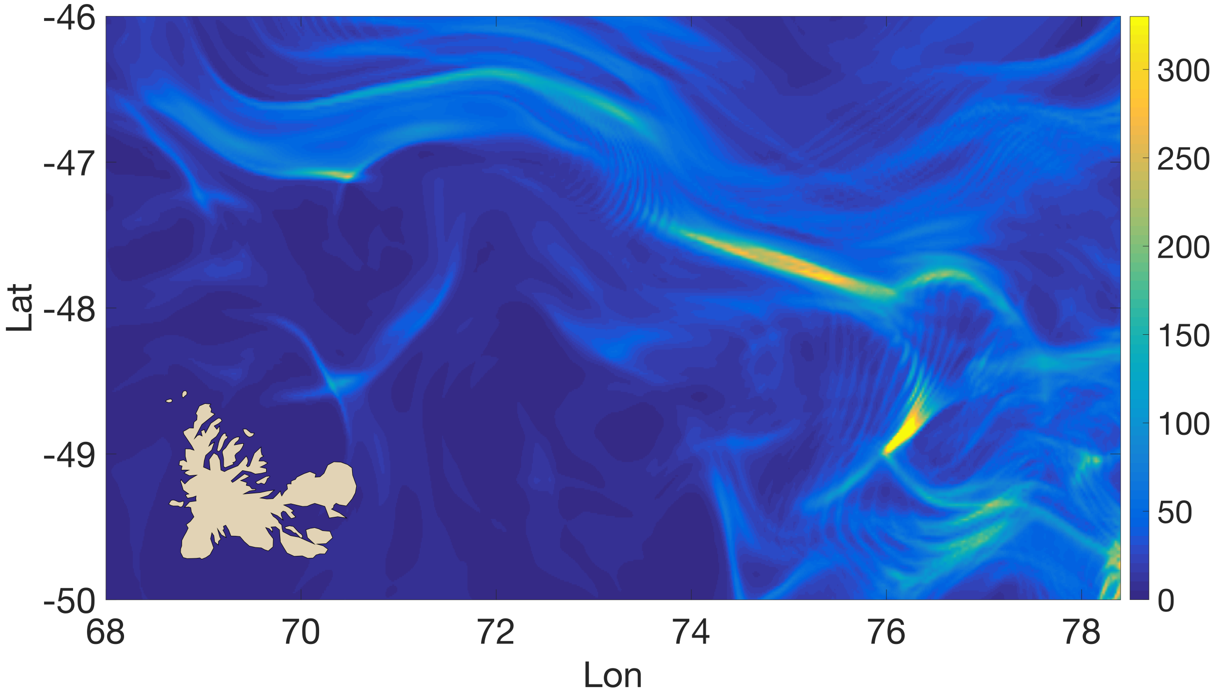

SUPPLEMENTARY FIG. 6: B^L field in the same region of Fig. 6 but for $\tau = 15$ (top panel) and 25 (bottom panel) from altimetry data. The strip location is persistent and we find small variations in its intensity.
