# Supplementary figures and images for: Lagrangian betweenness as a measure of bottlenecks in dynamical systems with oceanographic examples
Source: Nat Commun. 2021 Aug 16;12:4935. doi: 10.1038/s41467-021-25155-9 (PMC8368092; doi:10.1038/s41467-021-25155-9)

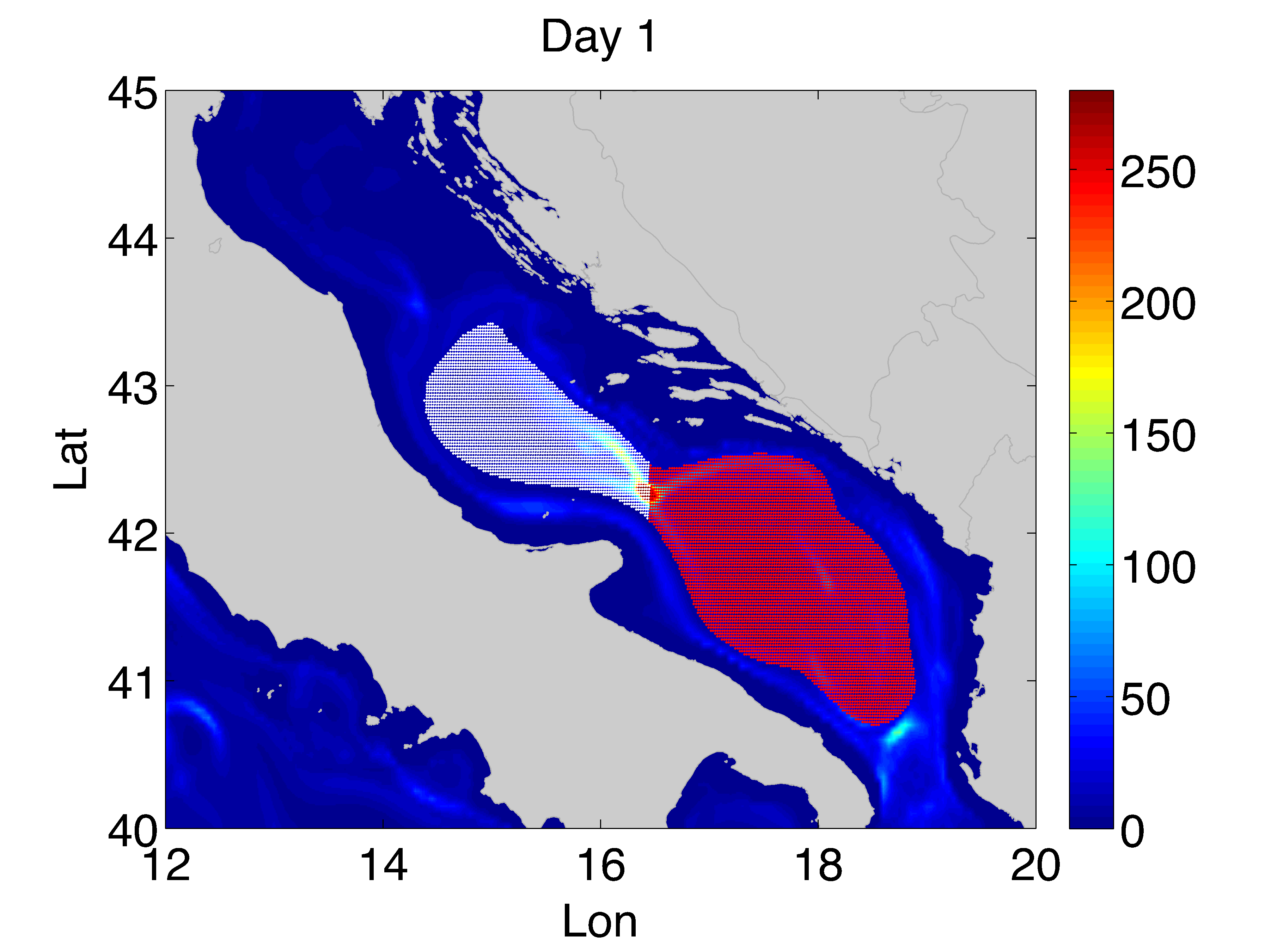

Supplement: Supplementary file 3 — Supplementary Movie 1 [file 41467_2021_25155_MOESM3_ESM.gif]

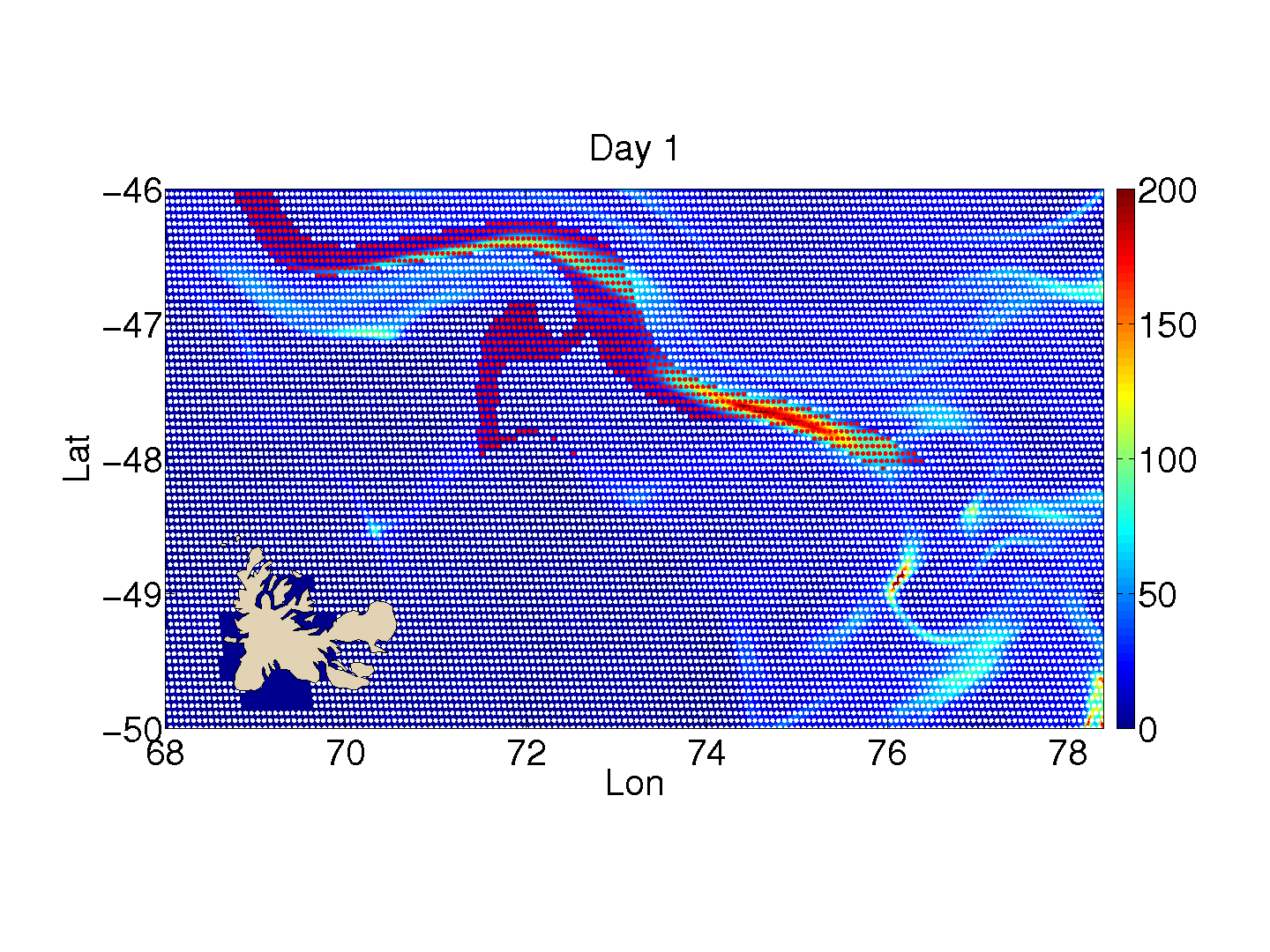

Supplement: Supplementary file 4 — Supplementary Movie 2 [file 41467_2021_25155_MOESM4_ESM.gif]
