# Supplementary material for: Lagrangian betweenness as a measure of bottlenecks in dynamical systems with oceanographic examples
Source: Nat Commun. 2021 Aug 16;12:4935. doi: 10.1038/s41467-021-25155-9 (PMC8368092; doi:10.1038/s41467-021-25155-9)
Supplement: Supplementary file 5 — Description of additional supplementary files [file 41467_2021_25155_MOESM5_ESM.docx]

Description of additional supplementary items

Title: Supplementary Movie 1

Description: Evolution of Lagrangian particles in the Adriatic Sea from the 1st to the 15th of December 2013 superimposed to the corresponding Lagrangian betweenness field.'

Title: Supplementary movie 2

Description: 'Evolution of Lagrangian particles in the Kerguelen region from the 1st to the 20th of December 2007 superimposed to the corresponding Lagrangian betweenness field.'
